# Extra, Extra, read all about it!

**DOI:** 10.1186/s42155-020-00192-5

**Published:** 2021-01-12

**Authors:** Jim A. Reekers

**Affiliations:** Professor Emeritus Interventional Radiology, Amsterdam UMC University of Amsterdam, Amsterdam, Netherlands

These are strange times. We are no longer able to go to scientific meetings to learn, discuss, be inspired and meet colleagues and friends. In the meantime, new communication tools have emerged, and it is amazing to see how fast these innovative communication platforms have shaped a new virtual world. Some of these groundbreaking virtual meetings are amazingly good, like the virtual CIRSE summit this year. An unexpected finding is that the number of subscribers to virtual meetings is much higher compared to physical meetings. I wonder what will remain of this new world when we have conquered the pandemic.

Meanwhile there is another scientific revolution going on, completely independent from Covid-19. This revolution is silent and will change our scientific world permanently. I am referring to the way we have been communicating science over the last 350 years. The first academic journals were the Journal des Sçavans followed soon after by Philosophical Transactions of the Royal Society (1665). The first fully peer-reviewed journal was Medical Essays and Observations (1733). The way we are communicating science and the process of peer-review has a long tradition. Around 2000, BioMed Central (now part of Springer Nature) was founded as the first and largest open access scientific publisher. Parallel to the development of information and communications technology (ICT) and the internet, open access publishing has developed very rapidly in the last two decades, next to the traditional subscription journals. Almost all subscription journals have now established a whole family of open access platforms next to the old “mother” journal; and parallel to this, stand-alone open access journals emerge monthly.

The concept behind open access was already promoted by the American sociologist Robert King Merton who declared in 1942, “Each researcher must contribute to the ‘common pot’ and give up intellectual property rights to allow knowledge to move forward.” I personally think that altruistic motives have little to do with this revolution of open access. It is a contemporary business model that follows online shopping, the internet, social media and the strive for inclusivity. It will only be a matter of time before all scientific publication is open access. The answer to the question if this will improve communication, transparency and inclusivity is not so clear yet – that all has to do with the nature of scientific publishing.

Scientific publishing is not about a scientific truth but about scientific facts. Truth is always contingent on historical and social context rather than being absolute and universal, and that truth is always partial and “at issue” rather than being complete and certain. This is what real science should be; always uncertain and open for discussion, embracing new facts and ideas. Science is not the truth, it is the pathway searching for the truth, which also explains the fact that we call it re-search. Recent political developments show that the truth is volatile but the facts are not, and the only way around this dilemma of alternative truth is to base the new truth on alternative facts. In science, alternative facts were always marked as fraud, a deadly sin in publishing science, often followed by expulsion from the scientific community. One of the ways to prevent scientific fraud has always been the peer-review process, already in place for more than 150 years. Although not 100% watertight, it is the best we have. There has always been criticism on peer-review, being non-transparent, patronizing and prejudiced, and this critique is often understandable. Therefore, the introduction of open peer-review in some new open access journals, like in CVIR Endovascular, is an important and irreversible development. It has many advantages, taking away the old criticism, and in my opinion making the published paper stronger and much more interesting, as the peer-review and the comments by the authors are now an integral part of the published paper. (Reekers, 2020) Until very recently I had no doubt that the shift from subscription journals with closed peer-review to open access with open peer-review is an unavoidable and irreversible next step in publishing science, but recent developments have made me very uncertain about this.

In the last couple of years, a new way to publish scientific data has emerged, and this is expanding very fast. I was shocked that many off my colleagues have not yet heard of this, so called, pre-print publishing. Pre-print is actually a platform where you can upload your scientific manuscript for everybody to read. It sounds like those digital platforms where you can upload your own music. But what really worries me is that if you visit one of those pre-print websites, the layout of the paper looks very real, like a medical paper as we all know it. And although, in fine print, it says that the manuscript is not peer-reviewed, the later is easily overlooked. But even more frightening is that these papers also get a permanent DOI number and are therefore citable and can be found through Google Scholar and Crossref. Interesting is also that journal publishers do not count pre-print as a reason to deny or disenfranchise a submission to their journal. Moreover, these manuscripts stay on these sites forever, and can also not be removed after they have been published in a peer-reviewed journal. I did a small random sample of pre-print papers published on these sites, and I could not find any paper that was also published in a peer-reviewed journal later on. I admit my random sample is not real science, but it is for me an indication that we are in a process of accepting and incorporating, non-scrutinized, potentially alternative scientific facts as part of the official scientific record.

With the speed of multiplication on the digital highway, I predict that we will soon have a parallel scientific world with non-peer-reviewed scientific manuscripts, on pre-print sites, containing references from other pre-print articles. When this happens, the scientific truth will be based on non-scrutinized, alternative scientific facts. This will lead to a complete collapse and inflation of science as we know and trust it today. The reality that this is not an unrealistic doom scenario was proven by the current president of the US who has recently touted that hydroxychloroquine is an effective drug to treat Covid-19, referring to a paper on a pre-print site as the scientific proof for his claims. Now 6 months later, this very paper still only exists in pre-print. If we, as doctors, have to base our medical decisions on alternative and non-scrutinized “scientific facts,” we will become, gradually and unnoticed, a bunch of 2.0 quacks, because every medical treatment without good scientific data is no more than a medical experiment.

## Data Availability

Not applicable.
